# Evaluation of Flavonoid Contents and Antioxidant Capacity of the Aerial Parts of Common and Tartary Buckwheat Plants

**DOI:** 10.3390/molecules17089668

**Published:** 2012-08-13

**Authors:** Danuta Zielińska, Marcin Turemko, Jacek Kwiatkowski, Henryk Zieliński

**Affiliations:** 1 Department of Chemistry, University of Warmia and Mazury in Olsztyn, Plac Łódzki 4, Olsztyn 10-727, Poland; 2 Department of Plant Breeding and Seed Production, University of Warmia and Mazury in Olsztyn, Plac Łódzki 3, Olsztyn 10-724, Poland; 3 Division of Food Science, Institute of Animal Reproduction and Food Research of Polish Academy of Sciences, Tuwima 10, P.O. Box 55, Olsztyn 10-747, Poland

**Keywords:** common buckwheat, tartary buckwheat, aerial parts, rutin, minor flavonoids, antioxidant capacity

## Abstract

The analysis of major and minor flavonoids, and antioxidant capacity of stems, leaves, flowers, unripe seeds and ripe seeds of common and tartary buckwheat plants collected during different growth periods was addressed in this study. The highest rutin contents were observed in flowers and leaves collected from common and tartary buckwheat at early flowering as well as flowering and seed formation states. A low quercetin contents were found in all studied aerial part of buckwheat plants. Quercitrin (quercetin-3-rhamnoside) was only found in flowers collected at different growth periods while flavone *C*-glucosides were accumulated preferentially only in unripe seeds collected from common buckwheat at an early flowering state. The rank of antioxidant capacity provided for aerial parts of common and tartary buckwheat at early flowering state was as follows: flowers > leaves > stems. The highest contribution of rutin to the antioxidant capacity of the aerial parts of common and tartary buckwheat was found for stems followed by leaves, flowers and unripe seeds. The results demonstrate that flowers from common and tartary buckwheat collected at early flowering as well as flowering and seed formation states have the future potential to be a useful food ingredient.

## 1. Introduction

Buckwheat, frequently classified as a pseudocereal, is one of the crops cultivated by various ethnic groups in developed and developing countries and an integral part of their diet and culture [1]. Among many species of buckwheat, common (*Fagopyrum esculentum*) and tartary buckwheat (*Fagopyrum tartaricum)* are of increasing interest [2], the latter being widely grown and utilized in western China. Many nutraceutical compounds found in tartary buckwheat are also suggested to have health benefits [3].

Buckwheat is considered to be a major dietary source of rutin (quercetin 3-rutinoside) as well as a minor source of other flavonoids. The antioxidant activity of rutin, quercetin and flavone *C*-glucosides was determined by different chemical assays and has been recently reported [4,5]. Moreover, antioxidative [6], antihypertensive [7] and anti-inflammatory activities [6] of buckwheat flavonoids were also demonstrated. Human studies of flavonoids have suggested effects that can in part be attributed to their antioxidant action [8].

Nowadays, healthiness is considered as one of the key drivers in food business, and both agricultural and pharmaceutical industry look for the natural flavonoids-rich material of high antioxidant capacity. Buckwheat herb as well as other plant extracts seems to be an attractive material for these requirements [9]. Up till now, the antioxidant activity of tartary (*F. tartaricum* (L.) Gaertn.) and common (*F. esculentum* Moench) buckwheat sprouts was reported [10], and only some reports are available on flavonoids in buckwheat seedlings focusing mostly on rutin [3,11,12,13,14].

The main objective of the present study was to quantify rutin and minor flavonoids in the aerial parts of common and tartary buckwheat (stems, leaves, flowers, unripe seeds and ripe seeds) as affected be the phenological state (early flowering, flowering, seed formation and ripening states). Moreover, a series of simple methodologies were evaluated that could be used as a tool for the selection of the aerial parts of buckwheat plant as food ingredient. Finally, the contribution of rutin to the antioxidant capacity of the aerial part of common and tartary buckwheat was also addressed. 

## 2. Results and Discussion

### 2.1. Dry Weight (DW) Content

Lyophilization, the selected drying method used in this study, was recently shown among others to have no effect on the chemical composition of buckwheat plant material [13]. DW content of aerial parts of buckwheat plants increased during the investigated growth period (data not shown). The lowest dry weight content was noted in stems at 41 DAS whereas the highest in ripe seeds at 100 DAS. DW was strongly depended on the phenological state of the plant. The higher DW contents were observed at flowering and seed formation states (48 DAS for common and 62 DAS for tartary buckwheat) than at early flowering (41 DAS). The aerial parts of tartary buckwheat plant showed higher DW contents when compared to respective parts of common buckwheat at the same phenological state. These results indicate that the flowering and seed formation states (48 DAS for common and 62 DAS for tartary buckwheat) are suitable for obtaining greater dry mass. The linear increase of the dry mass of leaves from 14 DAS to 42 DAS was also noted in common and tartary buckwheat varieties by Suzuki *et al*. [13].

### 2.2. Total Flavonoid Content (TF)

The TF content of the aerial parts of common and tartary buckwheat plant as affected by the phenological state are illustrated in Table 1.

The highest TF content was noted in common buckwheat flowers collected at flowering and seed formation states (204 mg RE/g DW) whereas 29% less was found in the respective flowers from tartary buckwheat (145 mg RE/g DW). About 50% less TF was noted in leaves of common and tartary buckwheat (54.8–81.9 mg RE/g DW) when compared to TF in flowers from early flowering, flowering and seed formation states. Stems (8.8–17.6 mg RE/g DW) and ripe seeds (5.8–20.2 mg RE/g DW) from both varieties were the poorest sources of TF. Interestingly, the TF contents of unripe seeds of common and tartary buckwheat were eight-fold and four-fold higher than the respective ripe seeds. The order of TF content for different plant parts of common and tartary buckwheat at early flowering state (41 DAS) was as follows: flowers > leaves > stems. The rank at flowering and seed formation state was: flowers > leaves > unripe seeds > ripe seeds > stems. Similar order (leaves > ripe seeds > stems) was also found by Bystrická *et al*. [14] when the content of total polyphenols was determined in different anatomical parts of common buckwheat plants of six cultivars. However, in the above study the growth period at which the plant material was harvested was not reported. Moreover, TPC was determined using the Folin-Ciocalteu reagent and results expressed as gallic acid equivalents on dry weight basis. Thus a direct comparison to the TF content provided in this study is not possible. Holasová *et al*. [15] also used common buckwheat seeds, hull, straws and leaves from mixed varieties for the analysis of total phenolics. They found half the level of total phenolics in leaves (39.5 mg GAE/g DW) when compared to the TPC content of leaves showed by Bystrická *et al*. [14]. Our data indicate that leaves, flowers and unripe flowers from common and tartary buckwheat could be considered as rich sources of flavonoids at 41–62 DAS (when the plants are in the flowering and seed formation state). Similar growth period was shown by Fabjan *et al*. [3] who harvested green material from tartary buckwheat plant from 50 DAS (flowering) to 63 DAS (first seed formation). Our finding support the suggestion by Fabjan *et al*. [3] that green buckwheat could be readily produced and used as a nutritionally rich material, a flavonoids-rich herb tea, or food additive due to its high polyphenol content. Similarly, Suzuki *et al*. [13] recommended the use of buckwheat leaves collected at 42 DAS as food ingredients, particularly in Ao-Jiru juice. Interest in flavonoids from buckwheat aerial parts has increased due to both their potential use as food ingredients as well as their impact on essential plant growth, development, stress adaptation and defense. In consequence, commercial interest in these compounds is considerable [16]. All of these aspects justify the intense interest in flavonoids, which has been manifested over several decades [17].

### 2.3. Rutin, Quercetin, Quercitrin and Flavone C-Glucosides Content

**Figure 1:**
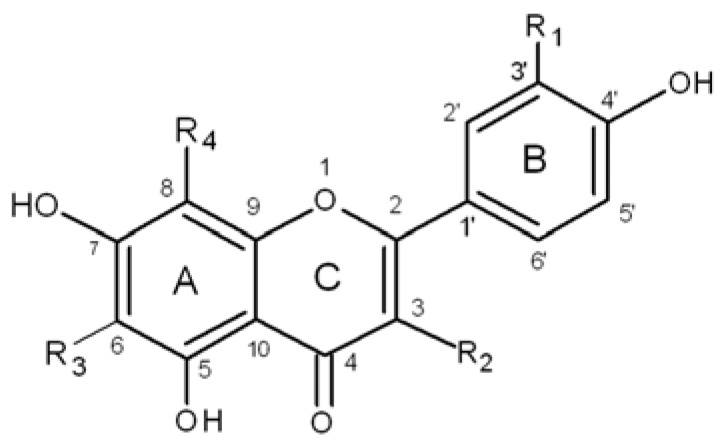
The chemical structure of buckwheat flavonoids.

Buckwheat is considered to be a major dietary source of rutin (quercetin 3-rutinoside), however, other flavonoids such as quercetin, myricetin, hyperoside (quercetin 3-O-β-D-galactoside), quercitrin (quercetin 3-*O*-α-L-rhamnoside), epicatechin and flavone *C*-glucosides were also identified in buckwheat during the last decade [2,3,18]. The main flavone *C*-glucosides present in buckwheat seeds include orientin and homoorientin, a pair of isomeric compounds, and their 4'-deoxy analogues, namely vitexin and isovitexin [19]. The chemical structure of the major buckwheat flavonoids is shown on Figure 1.

The rutin content in the aerial parts of common and tartary buckwheat are given in Table 2.

A significant effect of buckwheat variety and the phenological state was observed on rutin content in stems, leaves, flowers, unripe seeds and ripe seeds. The highest content of rutin was observed in leaves (5.1–8.2% DW) and in flowers (7.2–7.7% DW) collected from common and tartary buckwheat at early flowering (41 DAS) as well as flowering and seed formation states (48–62 DAS). The order of rutin content in different plant parts of common and tartary buckwheat at early flowering state (41 DAS) was as follows: flowers > leaves > stems. Similar order was found at flowering and seed formation states: leaves ≥ flowers > unripe seeds > stems. The buckwheat seeds were the poorest source of rutin. The rutin content noted in tartary buckwheat ripe seeds was 31-fold higher in comparison to its content in common buckwheat ripe seeds however it was at comparable levels to that found in stems at 41 DAS. Rutin content found in our study was half of that measured in the herb of spring-sown tartary buckwheat varieties China 1, China 2, and Lux by Fabjan *et al*. [3]. They reported content of rutin up to 3% DW when plant material was collected between 50 and 87 DAS. Moreover, content of rutin in leaves provided in our study was also half of that reported for the same material from common buckwheat by Holasová *et al*. [15] (2.3% DW) and by Bystrická *et al*. [14] (2.6–3.8% DW). In contrast, Suzuki *et al*. [13] noted higher content of rutin in 42 DAS leaves of tartary buckwheat Hokkai T10 (10% DW). Rutin content in stems was similar to earlier studies [14] (0.53–0.80% DW). These results also agree with those obtained by Kreft *et al*. [20]. 

It was reported by Kalinová and Dadáková [21] that common buckwheat flowers also contain high levels of rutin, however variety and environmental conditions may influence its concentration. Our findings, along with other studies [2,13,14,15,22] indicate that there is a wide variation of rutin content in buckwheat plant material depending on the species, variety, harvesting period and environmental conditions.

In this study low quercetin contents within the range of 0.01–0.25% DW were found in all studied aerial parts of buckwheat plants (data not shown). The highest quercetin content was noted in flowers from tartary buckwheat collected at 41 and 62 DAS (0.24–0.25% DW) but it was about 30-fold less than the rutin content in these parts. In common buckwheat, quercetin content found in leaves collected at early flowering state as well as at the flowering and seed formation state was about half (0.13–0.15% DW) in comparison to those found in flowers. This could be due to the hydrolysis of rutin by a recently described rutin hydrolyzing enzyme isolated from *Fagopyrum tataricum* Moench seeds [23]. The stems, unripe seeds and ripe seeds were the poorest source of quercetin; its content ranged from 0.001 to 0.012% DW for common buckwheat and from 0.045 to 0.086% DW for tartary buckwheat during the growth period. 

Quercitrin (quercetin-3-rhamnoside) was only found in flowers collected from common buckwheat plant at early flowering state (0.54 ± 0.03% DW) and at flowering and seed formation state (1.80 ± 0.10% DW). In our study no quercitrin was found in the aerial parts of tartary buckwheat, however concentrations within the range from 0.001 to 0.052% DW were reported in the herb of spring-sown tartary buckwheat varieties between 50 and 87 DAS [3]. Currently, there is no information related to the concentration of quercitrin in flowers from common and tartary buckwheat, however it might be possible that quercitrin may accumulate preferentially there. 

**Figure 2:**
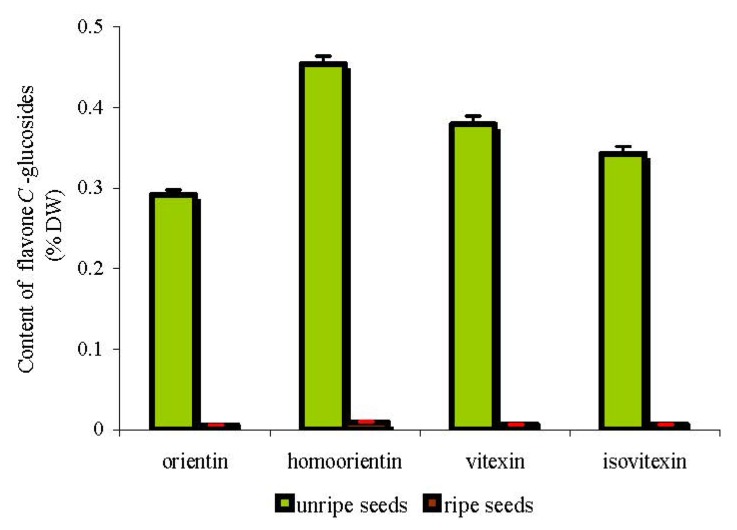
The content of flavone *C*-glucosides in the unripe and ripe seeds of common buckwheat harvested at 48 and 100 DAS, respectively (% DW).

In this study, flavone *C*-glucosides, namely orientin, homoorientin, vitexin and isovitexin, were only found in unripe and ripe seeds collected from common buckwheat at 48 and 100 DAS, respectively (Figure 2).

The unripe seeds were a significant source of flavone *C*-glucosides as their individual content exceeded almost 60-times those found in ripe seeds. This is in agreement to the observation made by Suzuki *et al*. [13,24] who did not detect flavone *C*-glucosides in leaves collected at 42 DAS from common and tartary buckwheat varieties, however they showed concentrations of flavone *C*-glucosides at trace level during seed maturation of common and tartary buckwheat as we also did in this study for common buckwheat ripe seeds. These minor flavonoids have received much attention because of their suggested antioxidant and anticancer properties [16]. Recently we showed the following rank of the antioxidant activity of flavone *C*-glucosides as compared to quercetin: quercetin ≥ homoorientin > orientin > isovitexin > vitexin [5]. Various biological and pharmacological activities have been attributed to these compounds, such as hypotensive, anti-inflammatory, antispasmodic [25], antimicrobial [26], radioprotective effects [27] and anti-glycation activities [28]. Therefore, preferentially accumulation of flavone *C*-glucosides in unripe seeds collected from common buckwheat at 48 DAS is an important outcome of this study.

### 2.4. Antioxidant Capacity (AC) Determined by DPPH and PCL Assays

The antioxidant capacity of the aerial parts of common and tartary buckwheat plant and buckwheat seeds determined against DPPH (DPPH^•^) and superoxide anion (O_2_^−•^) radicals are shown in Table 3 and Table 4, respectively.

Results indicated a wide range of AC values for stems, leaves, unripe and ripe seeds from common and tartary buckwheat collected during early flowering, and flowering and seed formation state. The highest AC values were noted for common and tartary buckwheat flowers collected at early flowering up to full flowering and unripe seed formation states. AC values of flowers from early flowering state (41 DAS) determined by DPPH and PCL assays were within the range of 420–577 µmol TE/g DW however those from flowering and unripe seed formation state showed even higher AC. The highest increase of AC by 24 and 81% was found for flowers from common buckwheat when results were provided by DPPH (Table 3) and PCL assay (Table 4), respectively.

The order of AC values for different plant parts of common and tartary buckwheat at early flowering state (41 DAS) was as follows: flowers > leaves > stems. Similar order was found at flowering and seed formation state: flowers > leaves > unripe seeds > stems. 

The leaves from tartary buckwheat collected at 41 DAS showed AC value 263 µmol Trolox/g DW (DPPH assay) and 427 µmol TE/g DW (PCL assay). These values were higher by 70% (DPPH assay) and 46% (PCL assay) than AC of those from common buckwheat (Table 3 and Table 4). Interestingly, contradictory findings were noted for leaves from flowering and seed formation state with the AC value being 10% (DPPH assay) and 42% (PCL assay) higher in the leaves from common buckwheat. The AC values of stems from common and tartary buckwheat were the lowest amongst the aerial parts and were independent of the phenological state. A drastic reduction of AC due to the ripening state at 100 DAS resulted in the lowest AC values in seeds. However, the AC of ripe seeds from tartary buckwheat when compared to those from the common variety was eight-fold higher.

There is a lack of a systematic study of antioxidant capacity of the aerial parts of common and tartary buckwheat using both methods applied in our study. Suzuki *et al*. [13] reported AC determined by DPPH assay of leaves collected from common and tartary buckwheat at 42 DAS about 200 µmol TE/g DW which was within the AC of leaves (154–262 µmol TE/g DW) reported in this study for leaves harvested at 41 DAS. Bystrická *et al*. [14] also evaluated the antioxidant capacity of stems, leaves and seeds from six common buckwheat cultivars however direct comparison of the results is complicated as antioxidant capacity was expressed as percentage of DPPH inhibition. Holasová *et al*. [15] used the Schaal oven test for the determination of the antioxidant capacity of buckwheat seeds, straws and leaves expressed as protection factor. Based on the studies performed by Bystrická *et al*. [14] and Holasová *et al*. [15], the quality rank for AC (leaves > stems > seeds) was confirmed in our study for the same parts of common and tartary buckwheat plants. The AC values of the aerial parts of common buckwheat plant determined against DPPH^•^ and O_2_^−•^ were positively correlated (r = 0.94). The weaker correlation was found for AC values of aerial parts from tartary buckwheat (r = 0.80). Both applied assays for AC determination may serve as a useful tool for screening the aerial parts of buckwheat for antioxidant capacity. 

### 2.5. The Contribution of Rutin to the Antioxidant Capacity of Buckwheat Plant Material

**Figure 3:**
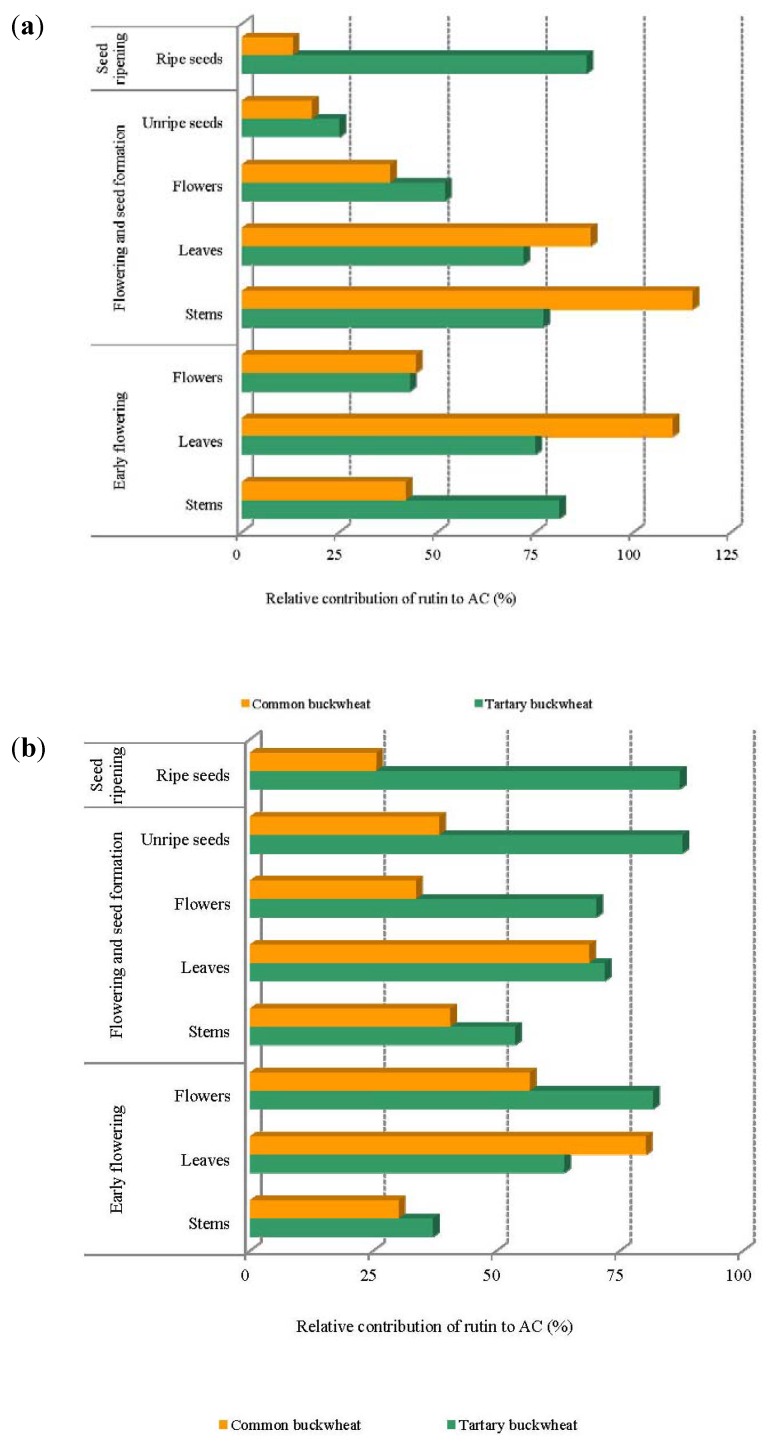
The contribution of rutin to the antioxidant capacity of the aerial parts of common and tartary buckwheat provided by: (**a**) DPPH assay; (**b**) PCL assay.

Rutin content in the aerial part of common and tartary buckwheat plants was positively correlated with AC evaluated by the DPPH (r = 0.81 and r = 0.90, respectively) and photochemiluminescence assay (r = 0.88 and r = 0.97, respectively), thus providing a good background for the calculation of the contribution of rutin to the antioxidant capacity of buckwheat plant material. The calculation was based on the previously published antioxidant activity of rutin provided by DPPH (2.03 ± 0.01 mM Trolox) and PCL (1.38 ± 0.07 mM Trolox) [4]. The contribution of rutin to the antioxidant capacity determined by means of DPPH and PCL assays is shown on Figure 3, respectively.

The highest contribution of rutin to the antioxidant capacity of the aerial parts of common and tartary buckwheat evaluated by DPPH method was found for stems followed by leaves, flowers and unripe seeds (Figure 3a). Interestingly, the contribution of rutin to the antioxidant capacity of tartary buckwheat seed was higher than those found for other parts, thus confirming that tartary buckwheat seed is an important source of dietary rutin [3]. Rutin contribution to AC of leaves at 41 DAS and stems at 48 DAS from common buckwheat exceeded 100%. This could be due to the synergistic action of phenolics in 80% aqueous methanol extracts however this was not observed in the same aerial plant material from tartary buckwheat. In addition to rutin found in leaves collected at 42 DAS, Suzuki *et al*. [13] showed the presence of γ-aminobutyric acid, 2"-hydroxynicotianamine, chlorogenic acid and anthocyanins, which may add an additional pool of antioxidant activity. The results showed about 50% contribution of rutin to the antioxidant capacity of flowers from common and tartary buckwheat, which were shown in this study as the richest source of rutin followed by quercetin and quercitrin. These finding clearly indicate that in buckwheat flowers about half of the AC origins from the antioxidant activity of quercetin, quercitrin and other unidentified compounds. A similar trend in rutin percentage contribution to AC evaluated by PCL was noted (Figure 3b). It is worthily to note that rutin contribution to AC of all aerial parts from tartary buckwheat was higher when compared to the common buckwheat with one exception made to leaves harvested at early flowering stage. The highest contribution, around 87%, was found for unripe and ripe seeds of tartary buckwheat while that found for common buckwheat seeds did not exceed 25%. This was consistent with the rutin content shown in Table 2. The bitter taste of tartary buckwheat seeds is ascribed mainly to rutin, and since common buckwheat contains much less rutin, it has become more popular due its better taste [3]. Fortunately, buckwheat flowers were found to be the richest source of rutin and minor flavonoids in this study. However, due to the high rutin contents any future application of buckwheat flowers as a food ingredient will require sensory analysis of the enriched products and feeding experiments on animal and humans to prove any health promoting effects.

## 3. Experimental 

### 3.1. Chemicals

2,2-Diphenyl-1-picrylhydrazyl (DPPH), 6-hydroxy-2,5,7,8-tetramethylchroman-2-carboxylic acid (Trolox), quercetin, rutin (quercetin-3-rutinoside) were purchased from Sigma (Sigma Chemical Co., St. Louis, MO, USA). Quercitrin (quercetin-3-rhamnoside), orientin (3',4',5,7-tetrahydroxyflavone-8-*C*-glucoside), homoorientin (3',4',5,7-tetrahydroxy-flavone-6-*C*-glucoside), vitexin (4',5,7-trihydroxyflavone-8-*C*-glucoside) and isovitexin (4',5,7-trihydroxyflavone-8-*C*-glucoside) of HPLC-grade were obtained from Extrasynthese Company Inc. (Lyon, France). Photochemiluminescence analytical kit for measurement of the antioxidant capacity (kit No. 400.801) was from Analytik Jena AG (Jena, Germany). Methanol, acetonitrile, formic acid, acetic acid (supra-gradient) and sodium acetate were from Merck KGaA, Darmstadt, Germany. All other reagents of reagent-grade quality were from POCh (Gliwice, Poland). Water was purified with a Milli-Q-system (Millipore, Bedford, OH, USA). All solutions prepared for HPLC were filtered through a 0.45 µm nylon filter before use.

### 3.2. Buckwheat Plant Material

The common buckwheat cv ‘Volma’ (from the Belarusian plant breeding program) and tartary buckwheat variety (local accession from the Olsztyn region, Poland) were tested. They were grown at the experimental field of the Production-Experimental Station of the University of Warmia and Mazury in Olsztyn situated in North-East Poland. During the vegetation period there was no mechanical or chemical treatment. The leaves, stems, flowers, unripe seeds and ripe seeds were harvested in three states: at early flowering after 41 days after sowing (DAS), at flowering and seed formation after 48 (common buckwheat) and 62 DAS (tartary buckwheat), and at seed ripening after 100 DAS. Ten randomly selected plants per each growth state were collected to obtain enough aerial parts of both types of buckwheat. The plant material was lyophilized directly just after harvesting and the dried stems, leaves, flowers and seeds were ground individually to a fine powder and stored at −40 °C until chemical analysis.

### 3.3. Extraction

Methanol extracts were prepared as follows: milled and dried samples (approximately 100 mg) were accurately weighed and extracted with 80% aqueous methanol (1 mL) at room temperature. The mixture was vortexed for 30 s, sonicated for 30 s and centrifuged for 5 min (13,200 ×*g* at 4 °C). The extraction procedure was repeated five times and supernatants were combined and collected in a 5 mL volumetric flask and the total volume adjusted up to 5 mL. All extracts were kept at −40 °C prior to further analysis.

### 3.4. Determination of Total Flavonoids (TF)

TF content was determined with a colorimetric method according to [29]. Briefly, 80% methanol plant extract (0.25 mL) was diluted with distilled water (1.25 mL). Then a 5% NaNO_2_ solution (75 µL) was added, and the mixture was left at room temperature. After 6 min, a 10% AlCl_3_ × 6 H_2_O solution (150 µL) was added, and the mixture was allowed to stand for another 5 min. After that, 1 M NaOH (0.5 mL) was added. The solution was thoroughly mixed, and the absorbance was measured immediately against the prepared blank sample at 510 nm using a spectrophotometer (UV-160 1PC, Shimadzu, Kyoto, Japan). Total flavonoids content was standardized against rutin and the results (means and standard deviation for three independent extractions) were expressed as mg of rutin equivalents (RE)/g DW (dry weight). The linearity range for this assay was determined at 0.06–1.0 mg/mL (R^2^ = 0.99).

### 3.5. Quantitative Determination of *Flavonoids by* High-Performance Liquid Chromatography with Diode Array Detection (HPLC-DAD)

The 80% methanol plant extracts were subjected to identification and quantitative analysis of flavonoids using an HPLC system (Shimadzu), consisting of two pumps (LC-10 AD), DAD detector (SPD-M10A_VP_) set at 330 nm, autosampler set to 20 µL injection (SIL-10 AD_VP_), column oven (CTO-10 AS_VP_) and system controller (SIL-10 AD_VP_) (HPLC-DAD). All chromatographic analysis were performed at 35 °C on C18(2) Luna 5 µm column, 4.6 × 200 mm (Phenomenex, Torrance, CA, USA) and at a flow rate of 0.8 mL/min. The flavonoids were eluted using aqueous 4% formic acid (solvent A) and acetonitrile containing 4% of formic acid (solvent B). The gradient elution programme was as follows: 12-22-70-12-12% B at gradient time t_G_ = 0 − 9 − 22 − 40 − 45 − 50 min. Rutin, quercetin, quercitrin, orientin, homoorientin, vitexin and isovitexin stock solutions were prepared in methanol at the concentration of 500, 500, 500, 517, 477, 509 and 574 µM, taking into account the purity of the standards. For quantitative analysis, calibration standards were prepared in duplicate at five concentrations within the range of 0.1–40 µM of each compound. The content of flavonoids in the aerial parts of common and tartary buckwheat plants was expressed as percentage of DW. All data are the average of three independent extractions.

### 3.6. DPPH Radical Scavenging Assay

The DPPH^•^ scavenging activity was determined according to [19]. The scavenging activity of 80% methanol plant extracts and Trolox standard solutions (concentration range of 0.1–2.5 mM) in 80% methanol was assayed under the same conditions. The DPPH^•^ scavenging capacity of 80% methanol plant extracts was expressed in terms of Trolox equivalents based on the decrease in the absorbance of the DPPH^•^ solution as affected by the standards at 515 nm. Measurements were carried out using a UV-160 1PC temperature-controlled spectrophotometer with a CPS-Controller (Shimadzu). Total antioxidant capacity was expressed as µmol Trolox equivalents (TE)/g DW.

### 3.7. Photo-Induced Chemiluminescence Assay

The photo-induced chemiluminescence (PCL) assay, carried out using the method proposed by Popov [30], was performed to measure the antioxidant capacity of buckwheat plant extracts against superoxide anion radicals (O_2_^−•^) generated from luminol, a photosensitizer, under exposure to UV light. The antioxidant capacity of 80% methanol plant extracts was determined using analytical kits supplied by Analytik Jena (Leipzig, Germany). Measurements were performed with a Photochem^®^ apparatus (Analytik Jena) as reported previously [31]. The antioxidant capacity was expressed in µmol TE/g DW. 

### 3.8. Contribution of Rutin to the Antioxidant Capacity of Buckwheat Plant Material

The contribution of rutin to the antioxidant capacity of the extracts of the aerial parts of buckwheat with the highest AC value was calculated based on the rutin antioxidant activity as provided by DPPH and PCL. The antioxidant capacity was expressed in µmol Trolox equivalents (TE)/g DW. The rutin content of the extracts as determined by HPLC was converted into µmol/g DW, multiplied by its antioxidant activity, divided by the antioxidant capacity of the aerial part of buckwheat plants and expressed as percentage of contribution [4].

### 3.9. Statistical Analysis

Results of the chemical analyses are given as mean values and the standard deviation of nine independent measurements. The results were subjected to one-way analysis of variance (ANOVA) using Fisher’s Least Significant Difference (LSD) test and the significant differences (*p* < 0.05) were calculated. Correlation analysis was also performed and the Pearson correlation coefficient is reported.

## 4. Conclusions 

In this study we showed the highest rutin contents in flowers (7.2–7.7% DW) and leaves (5.1–8.2% DW) collected from common and tartary buckwheat at the early flowering as well as flowering and seed formation states. Low quercetin contents within the range of 0.01–0.25% DW were found in all studied aerial parts of buckwheat plants. Quercitrin was only found in flowers collected from common buckwheat plant at the early flowering state (0.54 ± 0.03% DW) and at flowering and seed formation state (1.80 ± 0.10% DW) while flavone *C*-glucosides were accumulated preferentially only in unripe seeds collected from common buckwheat at early flowering state. The order of AC values at early flowering state was as follows: flowers > leaves > stems while at flowering and seed formation state: flowers > leaves > unripe seeds > stems. The positive correlation between results obtained by DPPH and PCL assays indicates that both methods may serve as a useful tool for selection of the aerial part of buckwheat with the highest antioxidant capacity. The highest contribution of rutin to AC of the aerial parts of common and tartary buckwheat was found for stems followed by leaves, flowers and unripe seeds. These results indicate that, in term of rutin content and its contribution to AC, flowers collected from common and tartary buckwheat at early flowering as well as flowering and seed formation states have the future potential to be a useful food ingredient.

## Figures and Tables

**Table:** 

**Buckwheat Flavonoids**	**R_1_**	**R_2_**	**R_3_**	**R_4_**
rutin	OH	rutinose	H	H
quercetin	OH	OH	H	H
quercitrin	OH	rhamnose	H	H
orientin	OH	H	H	glucose
homoorientin	OH	H	glucose	H
vitexin	H	H	H	glucose
isovitexin	H	H	glucose	H

**Table 1:** Phenological state and total flavonoids (TF) content in the aerial parts of common (CB) and tartary (TB) buckwheat.

DAS	Phenological State	Aerial Part	TF (mg RE/g DW)	
			Common Buckwheat	Tartary Buckwheat
41 (CB) 41 (TB)	early flowering	stems leaves flowers	10.53 ± 1.03 ^aA^ 54.80 ± 2.54 ^bA^ 142.98 ± 5.81 ^dB^	17.63 ± 1.88 ^aB^ 81.08 ± 9.44 ^bB^ 131.32 ± 2.37 ^cA^
48 (CB) 62 (TB)	flowering and seed formation	stems leaves flowers unripe seeds	8.82 ± 0.92 ^aA^ 81.89 ± 4.00 ^cA^ 203.63 ± 24.18 ^eA^ 47.80 ± 0.40 ^bA^	10.20 ± 1.55 ^aA^ 76.40 ± 16.10 ^bA^ 145.40 ± 28.75 ^cA^ 73.34 ± 1.15 ^bB^
100 (CB) 100 (TB)	seed ripening	ripe seeds	5.78 ± 0.23 ^aA^	20.24 ± 0.31 ^aB^

Data expressed as means ± standard deviations of three independent extractions (n = 3). Means in a column followed by the different lower case letter correspond to significant differences (*p* < 0.05). Means in the same raw followed by capital letter correspond to significant differences (*p* < 0.05).

**Table 2:** Phenological state and rutin content (% DW ± SD) in the aerial parts of common (CB) and tartary (TB) buckwheat.

DAS	Phenological State	Aerial Part	Rutin (% DW)	
			Common Buckwheat	Tartary Buckwheat
41 (CB) 41 (TB)	early flowering	stems leaves flowers	0.422 ± 0.048 ^aA^ 5.117 ± 0.461 ^cA^ 7.285 ± 0.248 ^dA^	1.183 ± 0.166 ^aB^ 5.924 ± 0.070 ^cB^ 7.485 ± 0.248 ^dA^
48 (CB) 62 (TB)	flowering and seed formation	stems leaves flowers unripe seeds	0.763 ± 0.167 ^aA^ 8.237 ± 0.660 ^eB^ 7.761 ± 1.064 ^deA^ 1.573 ± 0.063 ^bA^	0.961 ± 0.014 ^aA^ 6.063 ± 0.832 ^cA^ 7.772 ± 0.993 ^dA^ 3.641 ± 0.202 ^bB^
100 (CB) 100 (TB)	seed ripening	ripe seeds	0.043 ± 0.004 ^aA^	1.350 ± 0.087 ^aB^

Means in a column followed by the different lower case letter correspond to significant differences (*p* < 0.05). Means in the same raw followed by capital letter correspond to significant differences (*p* < 0.05).

**Table 3:** Phenological state and antioxidant capacity (AC) of the aerial parts of common (CB) and tartary (TB) buckwheat provided by DPPH assay.

DAS	Phenological State	Aerial Part	AC (µmol TE/g DW)	
			Common Buckwheat	Tartary Buckwheat
41 (CB) 41 (TB)	early flowering	stems leaves flowers	33.39 ± 8.16 ^aA^ 154.23 ± 8.33 ^bA^ 543.72 ± 5.38 ^dA^	48.62 ± 9.40 ^aA^ 262.71 ± 16.51 ^bB^ 577.05 ± 7.63 ^eB^
48 (CB) 62 (TB)	flowering and seed formation	stems leaves flowers unripe seeds	21.95 ± 1.36 ^aA^ 307.08 ± 34.15 ^cA^ 675.77 ± 49.10 ^eB^ 285.68 ± 5.26 ^cA^	41.67 ± 4.93 ^aB^ 278.77 ± 40.51 ^bA^ 492.40 ± 30.82 ^dA^ 382.60 ± 4.10 ^cB^
100 (CB) 100 (TB)	seed ripening	ripe seeds	11.70 ± 0.97 ^aA^	50.67 ± 2.89 ^aB^

Data expressed as means ± standard deviations of three independent extractions (n = 3). Means in a column followed by different lower case letter correspond to significant differences (*p* < 0.05). Means in the same raw followed by capital letter correspond to significant differences (*p* < 0.05).

**Table 4:** Phenological state and antioxidant capacity (AC) of the aerial parts of common (CB) and tartary (TB) buckwheat provided by PCL assay.

DAS	Phenological State	Aerial Part	AC (µmol TE/g DW)	
			Common Buckwheat	Tartary Buckwheat
41 (CB) 41 (TB)	early flowering	stems leaves flowers	64.3 ± 8.8 ^abA^ 292.4 ± 66.6 ^cA^ 589.4 ± 3.2 ^dA^	146.4 ± 16.5 ^cB^ 426.9 ± 18.8 ^aB^ 420.1 ± 17.2 ^aB^
48 (CB) 62 (TB)	flowering and seed formation	stems leaves flowers unripe seeds	86.3 ± 15.4 ^abA^ 549.4 ± 54.7 ^dA^ 1065.9 ± 212.9 ^eA^ 187.9 ± 9.2b ^cA^	82.1 ± 9.1 ^bA^ 387.0 ± 46.9 ^aB^ 508.3 ± 71.40 ^dB^ 190.4 ± 6.2 ^cA^
100 (CB) 100 (TB)	seed ripening	ripe seeds	8.6 ± 0.2 ^aA^	71.1 ± 2.0 ^bB^

Data expressed as means ± standard deviations of three independent extractions (n = 3). Means in a column followed by the different lower case letter correspond to significant differences (*p* < 0.05). Means in the same raw followed by capital letter correspond to significant differences (*p* < 0.05).
